# Inflammatory and Immune Biomarkers in Mood Disorders: From Mechanistic Pathways to Clinical Translation

**DOI:** 10.3390/cells14191558

**Published:** 2025-10-08

**Authors:** Mario Pinzi, Andrea Fagiolini, Despoina Koukouna, Giacomo Gualtieri, Maria Beatrice Rescalli, Caterina Pierini, Simone Pardossi, Benjamin Patrizio, Alessandro Cuomo

**Affiliations:** Department of Molecular Medicine, School of Medicine, University of Siena, 53100 Siena, Italy; koukounadespoina@gmail.com (D.K.); giacomo.gualtieri2@unisi.it (G.G.); m.rescalli@student.unisi.it (M.B.R.); c.pierini@student.unisi.it (C.P.); s.pardossi@student.unisi.it (S.P.); benjamin.patrizio@student.unisi.it (B.P.); alessandro.cuomo@unisi.it (A.C.)

**Keywords:** neuroinflammation, mood disorders, biomarkers, precision psychiatry

## Abstract

Over the past two decades, immune–inflammatory dysregulation has emerged as a central paradigm in the biology of mood disorders. Patients with major depression (MDD) and bipolar disorder (BD) frequently display low-grade systemic inflammation. Elevated C-reactive protein (CRP), interleukin-6 (IL-6), and tumor necrosis factor-α (TNF-α) identify clinically relevant subgroups of patients characterized by greater severity, cognitive impairment, and poor treatment response. Changes in the gut microbiota and disruptions of the blood–brain barrier (BBB) act as important gateways through which systemic immune activity can influence the brain. At the intracellular level, pattern-recognition receptors activate convergent hubs including NF-κB, JAK/STAT, and MAPK cascades, while the NLRP3 inflammasome integrates mitochondrial dysfunction and oxidative stress with IL-1β release and pyroptosis. These pathways converge on glial dysregulation, impaired BDNF/TrkB signaling, and kynurenine pathway (KP) alterations, fostering excitotoxicity and synaptic deficits. Translational studies demonstrate that elevated CRP and IL-6 predict poor antidepressant outcomes. Anti-inflammatory agents such as infliximab and celecoxib show efficacy in specific subgroups of patients. Emerging multi-omics approaches identify immuno-metabolic biotypes, supporting the rationale for biomarker-guided stratification. These findings define an ‘inflammatory biotype’ of mood disorders and highlight the need for biomarkers and precision-based trials to guide treatment.

## 1. Introduction

The standard pharmacological treatments for major depressive disorder (MDD) and bipolar disorder (BD) fail to achieve remission in a considerable proportion of patients, many of whom continue to experience residual symptoms, chronic course, and marked functional impairment [1,2]. While the monoaminergic dysfunction model has long provided the dominant explanatory framework, it is now widely recognized as insufficient to account for the marked biological heterogeneity, variability in treatment response, and persistence of cognitive and affective symptoms observed in mood disorders [3,4]. Consequently, attention has shifted toward alternative pathophysiological models capable of integrating systemic and central mechanisms. Over the past two decades, converging lines of evidence have positioned immune–inflammatory dysregulation as a central dimension in the biology of mood disorders [5,6]. Meta-analyses consistently demonstrate low-grade systemic inflammation in both MDD and BD, with elevations in interleukin-6 (IL-6), tumor necrosis factor-α (TNF-α), and C-reactive protein (CRP) [7,8,9]. Notably, hs-CRP levels above 3 mg/L define a clinically relevant subgroup of patients characterized by heightened symptom severity, psychomotor slowing, cognitive impairment, and reduced responsiveness to antidepressants [9,10,11]. These findings suggest that mood disorders, at least in a biologically distinct subgroup, share features with chronic inflammatory syndromes, rather than representing conditions solely driven by neurotransmitter imbalance [6]. Crucially, inflammatory activity is not confined to peripheral soluble mediators but extends to several peripheral–central interfaces [12,13]. Alterations such as gut microbiota dysbiosis, reduced microbial diversity, depletion of butyrate-producing taxa, and enrichment of pro-inflammatory genera have been linked to altered short-chain fatty acid (SCFA) and indole metabolite production, as well as increased translocation of lipopolysaccharides (LPS) into circulation [14,15,16]. These changes amplify systemic immune activity and provide a mechanistic link between metabolic status and mood dysregulation [15,16]. Similarly, dysfunction of the blood–brain barrier (BBB) constitutes a critical interface: cytokines such as TNF-α and IL-6, together with reactive oxygen species, compromise tight junction integrity, allowing circulating inflammatory mediators to access the central nervous system (CNS) and foster neuroinflammation [12,13]. At the intracellular level, these peripheral signals converge on pattern-recognition receptors (PRRs) and downstream hubs. Toll-like receptor 4 (TLR4) recognizes microbial ligands such as lipopolysaccharides (LPS), while NOD1/2 receptors detect bacterial peptidoglycans, both of which can enter systemic circulation under conditions of bowel barrier dysfunction [14,17]. Engagement of these receptors activates nuclear factor kappa B (NF-κB), Janus kinase/signal transducer and activator of transcription (JAK/STAT), and mitogen-activated protein kinase (MAPK) pathways, thereby sustaining transcriptional programs of immune activation [18]. In parallel, the NLRP3 inflammasome links mitochondrial dysfunction, oxidative stress, and extracellular danger signals to IL-1β/IL-18 release and pyroptosis, establishing a further amplification loop [19,20,21]. These pathways ultimately converge on cellular effectors in the CNS, particularly microglia and astrocytes. Microglia and astrocytes, the primary immune regulators of the brain, become dysregulated under inflammatory conditions: hyperactive microglia release pro-inflammatory cytokines, glutamate, and reactive oxygen species, while impaired astrocytic function reduces trophic support and glutamate clearance [22,23]. Together with mitochondrial dysfunction and redox imbalance, these alterations foster excitotoxicity, energy failure, and progressive synaptic deficits [24,25]. Neurotrophic signaling is likewise disrupted, with cytokine-induced suppression of brain-derived neurotrophic factor (BDNF)/TrkB pathways contributing to dendritic spine loss and impaired synaptic plasticity [26,27]. Interestingly, rapid-acting antidepressants such as ketamine exert part of their therapeutic efficacy through rapid restoration of BDNF-dependent plasticity [28]. A further metabolic dimension is provided by the kynurenine pathway (KP), which represents a major biochemical interface between immune activation and neurotransmission [29,30]. Pro-inflammatory cytokines, particularly interferon-γ, TNF-α, and IL-6, activate indoleamine-2,3-dioxygenase (IDO) and tryptophan-2,3-dioxygenase (TDO), diverting tryptophan metabolism away from serotonin toward kynurenine and its neuroactive metabolites [29,31]. This shift results in increased production of quinolinic acid (QA), a neurotoxic NMDA receptor agonist, and reduced kynurenic acid (KYNA), a neuroprotective NMDA antagonist and α7 nicotinic receptor modulator, thereby contributing to excitotoxicity, impaired cognition, and pathology progression [32,33,34]. From a translational perspective, these mechanistic insights align with clinical evidence. Peripheral biomarker studies, neuroimaging with translocator protein positron emission tomography (TSPO-PET), metabolic assays, gut microbiome analyses, and interventional trials with anti-inflammatory and immunomodulatory agents all converge in supporting the existence of an “inflammatory biotype” of mood disorders [35,36]. Notably, clinical benefits are observed primarily in biomarker-enriched subgroups, underscoring the need for stratified and precision-based approaches [37,38]. Beyond immune–inflammatory dysregulation, mood disorders arise from the interplay of multiple vulnerability domains. Genetic factors contribute substantially, with genome-wide association studies implicating variants in genes regulating immune signaling, synaptic plasticity, and stress-response pathways [39]. Polygenic risk scores suggest that immune-related genetic architectures partly overlap with susceptibility to MDD and BD, reinforcing the biological plausibility of an immunogenetic interface [40]. Stress-related mechanisms represent another critical determinant. Chronic psychosocial stress activates the hypothalamic–pituitary–adrenal (HPA) axis, leading to hypercortisolemia, glucocorticoid receptor resistance, and sustained low-grade inflammation. These alterations potentiate the activation of NF-κB and NLRP3-dependent cascades, linking stress exposure to immune–inflammatory processes and to downstream effects on neuroplasticity and mitochondrial function [41]. Finally, environmental exposures—ranging from early life adversity and trauma to lifestyle factors such as diet, smoking, and physical inactivity—exert long-lasting effects on immune tone and brain health. Early adversity has been associated with persistent microglial priming and heightened inflammatory reactivity, while obesogenic diets and sedentary behavior promote systemic metabolic inflammation, further exacerbating neuropsychiatric vulnerability [6,42]. Collectively, these genetic, stress-related, and environmental dimensions interact with immune–inflammatory pathways, providing a multifactorial framework that captures the complexity and heterogeneity of mood disorders.

In this review, we synthesize findings from the past decade along four thematic domains. The first addresses peripheral triggers and barrier dysfunction, followed by intracellular signaling pathways, downstream final common pathways involving mitochondria, glia, plasticity, and kynurenine metabolism, and inter-individual differences in immune reactivity. The last section focuses on therapeutic implications and stratification approaches. By integrating these perspectives, we aim to outline an immune–inflammatory model of mood disorders that moves beyond descriptive associations toward mechanistic understanding and precision therapeutics.

## 2. Materials and Methods

We performed an extensive search using the following biomedical databases: Medline using the PubMed interface, Web of Science, and Embase. Search terms were as follows: “major depressive disorder”, “depression”, “bipolar disorder”, “bipolar depression” with immune–inflammatory and neurobiological concepts, including “cytokine”, “C-reactive protein”, “IL-6”, “TNF”, “neuroinflammation”, “translocator protein”, “kynurenine”, “blood–brain barrier”, “microbiome”, “oxidative stress”, “mitochondria”, and “BDNF”. Additional keywords were used for specific domains such as neuroimaging (TSPO-PET), kynurenine metabolites (quinolinic and kynurenic acid), cellular indices (NLR, PLR, MLR, monocyte subsets, T cells), intracellular signaling pathways (NF-κB, JAK/STAT, MAPK), inflammasome biology (NLRP3, IL-1β, IL-18), glial and astrocytic alterations, mitochondrial and redox pathways, synaptic plasticity, gut–brain axis, and BBB markers. No restriction on publication date was applied, and the last search was performed on 26 August 2025. Only articles written in or translated into English were considered. Priority was given to systematic reviews, meta-analyses, randomized controlled trials, large cohort studies, and mechanistic human investigations addressing immune–inflammatory biomarkers in mood disorders. Artificial intelligence was employed exclusively for linguistic polishing of the text. All aspects of the study—conception and design, data collection, analysis, interpretation, and conclusions—were carried out entirely by the authors, who take full responsibility for the integrity and accuracy of the work.

## 3. Mechanistic Pathways of Immune–Inflammatory Dysregulation in Mood Disorders

### 3.1. Peripheral Triggers and Barrier Dysfunction

**Figure 1:**
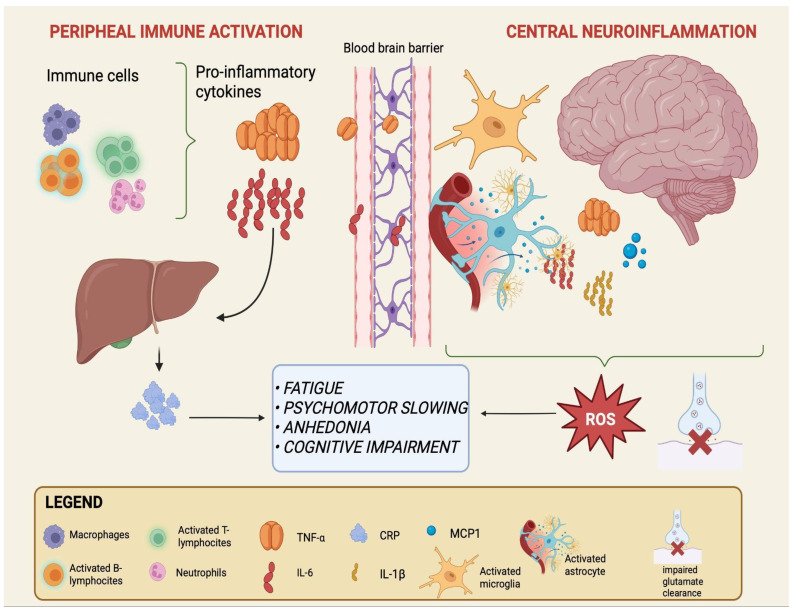
From peripheral immune activation to central neuroinflammation in mood disorders. Activated immune cells in the periphery (macrophages, neutrophils, and T- and B-lymphocytes) release pro-inflammatory cytokines, primarily IL-6 and TNF-α. IL-6 also stimulates hepatic production of CRP, a systemic inflammation marker. Increased cytokine load can compromise BBB integrity, facilitating their entry into the CNS. Within the brain, astrocytes and microglia become activated and release additional mediators, including IL-6, TNF-α, IL-1β, and MCP-1, while astrocytes show impaired glutamate clearance. This peripheral-to-central immune cascade sustains neuroinflammation and oxidative stress and is associated with core clinical symptoms of mood disorders such as fatigue, psychomotor slowing, anhedonia, and cognitive impairment. Created with BioRender (web-based version). Pinzi, M. (2025) https://BioRender.com/t7otmbn (accessed on 14 September 2025).

Peripheral triggers include a range of signals generated outside the CNS—such as microbial metabolites, dietary products, and systemic inflammatory mediators—that may contribute to the immune alterations observed in mood disorders [14,15,43]. While their precise role remains debated, a recurrent model suggests that these peripheral factors can shape systemic immune activity and, over time, influence the function of critical interfaces such as the intestinal epithelium and the BBB [13,15]. In this framework, gut dysbiosis and barrier permeability may represent early events, followed by immune activation and ultimately by neuroinflammatory changes that may contribute to the emergence of mood disorders, although the directionality and causality of these processes are not fully established [16,44]. The sequential processes linking peripheral immune activation to central neuroinflammation are schematically depicted in Figure 1.

#### 3.1.1. Gut–Brain Axis and Microbial Dysbiosis

**Figure 2:**
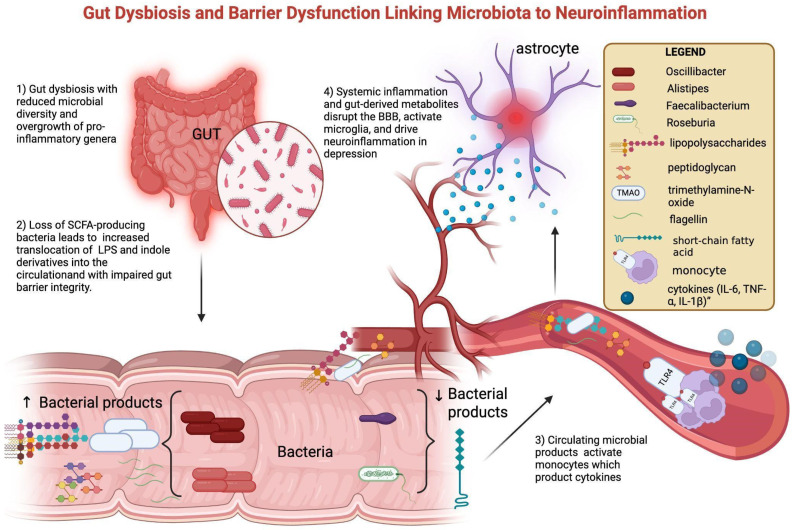
Gut dysbiosis and barrier dysfunction linking microbiota to neuroinflammation. Gut dysbiosis in mood disorders is characterized by reduced microbial diversity, with an overrepresentation of pro-inflammatory genera (Oscillibacter, Alistipes) and a reduction in SCFA-producing bacteria (Faecalibacterium, Roseburia). Loss of butyrate (a short-chain fatty acid, SCFA) weakens intestinal barrier integrity, while microbial products such as lipopolysaccharide (LPS), peptidoglycan (PGN), flagellin, and trimethylamine-N-oxide (TMAO) translocate into the circulation. These pathogen-associated molecular patterns activate circulating monocytes through pattern-recognition receptors, inducing the release of pro-inflammatory cytokines (IL-6, TNF-α, and IL-1β). Systemic inflammation and gut-derived metabolites promote BBB dysfunction and microglial activation, ultimately driving neuroinflammation and contributing to depressive symptomatology. Created with BioRender.com (web-based version). Pinzi, M. (2025) https://BioRender.com/blocq6n (accessed on 14 September 2025). Original figure created by the authors.

Alterations in the gut microbiota are among the best-studied contributors to peripheral immune–metabolic activation. Patients with MDD and BD frequently display reduced microbial diversity, characterized by enrichment of pro-inflammatory taxa such as *Alistipes* and *Oscillibacter*, and depletion of butyrate-producing genera including *Faecalibacterium* and *Roseburia* [45,46]. These compositional changes are associated with functional shifts: reduced SCFA production and increased translocation of lipopolysaccharides (LPSs) and indole derivatives into the circulation [14]. Loss of butyrate (a SCFA) weakens intestinal barrier integrity, while microbial products such as lipopolysaccharide (LPS), peptidoglycan (PGN), flagellin, and trimethylamine-N-oxide (TMAO) translocate into the circulation. Once in peripheral blood—following translocation across a compromised intestinal barrier—these molecules can interact with innate immune receptors, fostering the release of pro-inflammatory cytokines (IL-6, TNF-α, and IL-1β) and amplifying systemic immune activity. This peripheral immune activation may, over time, contribute to BBB dysfunction and facilitate neuroinflammatory processes [13,15,17]. Clinical studies confirm this link: elevated serum LPS and reduced SCFA correlate with higher CRP and IL-6 in MDD, while altered plasma indole-3-propionic acid associates with depressive severity in BD [47]. Multi-omics approaches show that microbial–metabolite signatures not only discriminate MDD from controls but also correlate with symptom severity [38]. Fecal microbiota transfer from depressed patients to germ-free mice induces depressive-like behaviors, supporting causality. Interventional trials with probiotics and prebiotics suggest modest mood improvements, often accompanied by CRP and IL-6 reductions, although with heterogeneous results [44,48]. Together, these data point to a gut-driven immune–metabolic depression subtype. Mechanistic interactions between gut dysbiosis, barrier dysfunction, and neuroinflammation are shown in Figure 2**.**

#### 3.1.2. Blood–Brain Barrier Dysfunction

Peripheral immune activation—whether originating from the gut or from other systemic sources—can in turn influence the BBB. Pro-inflammatory cytokines such as TNF-α and IL-6, together with ROS, have been shown to disrupt tight junction proteins, including occludin and claudin-5, leading to increased permeability [12,13]. This condition may permit cytokines, LPS, or other circulating inflammatory mediators to reach the CNS, where they can activate glial cells and foster neuroinflammation [14]. Several biomarkers support the relevance of BBB dysfunction in mood disorders. Elevated serum S100B—a glial-derived protein that enters circulation when barrier permeability is impaired—has been repeatedly reported in both MDD and BD, where higher concentrations correlate with illness severity and even predict poor antidepressant response, underscoring its potential as both a state and prognostic marker [49,50,51,52]. Increased levels of MMP-9, an enzyme that degrades extracellular matrix components, produced in response to inflammatory stimuli, have been associated with acute depressive episodes, while an elevated CSF/serum albumin ratio has been found in subsets of depressed patients, suggesting impaired barrier function [53,54]. Overall, these findings suggest that systemic inflammation and BBB alterations form a bidirectional loop, where peripheral immune signals compromise barrier integrity and, conversely, barrier dysfunction facilitates central immune activation and amplification of neuroinflammation through continuous peripheral-to-central signaling. Although the precise sequence remains unresolved, this model provides a plausible bridge linking systemic immune changes to neuroinflammation in mood disorders [38].

### 3.2. Intracellular Signaling Pathways Linking Peripheral Inflammation to the CNS

#### 3.2.1. Toll-like Receptors and NF-κB Activation

A central mechanism through which peripheral inflammatory signals influence the CNS involves the activation of pattern-recognition receptors (PRRs) located on the cell surface. Toll-like receptor 4 (TLR4), expressed on the plasma membrane, is responsive to lipopolysaccharides (LPS) and related microbial products that enter systemic blood following increased intestinal permeability [14,55]. In addition, damage-associated molecular patterns (DAMPs) such as HMGB1 or extracellular ATP, released during cellular stress, act as endogenous ligands that further activate TLR4 signaling [56].

Engagement of TLR4 initiates intracellular cascades involving NF-κB, JAK/STAT, and MAPK pathways. These hubs are also downstream of cytokine receptors activated by IL-6, TNF-α, and IL-1β. There is growing evidence implicating TLR4-related intracellular signaling pathways—particularly NF-κB, JAK/STAT, and MAPK cascades—as key mediators in the pathophysiology of mood disorders [57].

**Figure 3:**
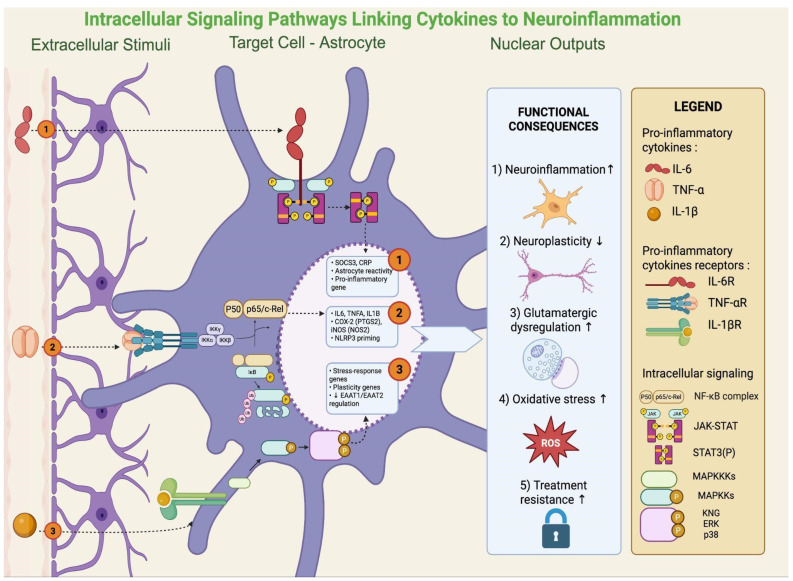
Intracellular cytokine signaling in astrocytes: pathways sustaining neuroinflammation and plasticity loss. Once bound to their receptors on astrocytes (IL-6R, TNFR, and IL-1β), cytokines activate three main signaling modules: (i) JAK/STAT3, promoting astrocyte reactivity and pro-inflammatory gene programs (e.g., SOCS3, CRP); (ii) NF-κB, inducing transcription of IL6, TNFα, IL1B, PTGS2 (COX-2), NOS2 (iNOS), and NLRP3 priming; and (iii) MAPK pathways (p38/ERK/JNK), regulating stress-response and plasticity-related genes, including EAAT1/2 transporters. Collectively, these cascades amplify neuroinflammation, impair neuroplasticity, disrupt glutamatergic homeostasis, increase oxidative/NO stress, and contribute to treatment resistance. Created with BioRender (web-based version). Pinzi, M. (2025) https://BioRender.com/jnzhwjy (accessed on 14 September 2025).

In rodent models, NF-κB activation in the hippocampus mediates stress-induced suppression of neurogenesis and depressive-like behavior [18]. In patients with MDD, increased NF-κB activity in peripheral blood mononuclear cells correlates with symptom severity and treatment resistance [18]. Persistent IL-6/STAT3 signaling has been linked to impaired neuroplasticity, while MAPK activation (p38, ERK, JNK) in microglia enhances glutamate release and ROS generation, promoting excitotoxicity [58]. Dysregulated MAPK signaling has been confirmed in postmortem corticolimbic tissue of depressed patients [59]. Activation of these intracellular pathways ultimately results in the production and release of pro-inflammatory cytokines, a phenomenon that has been consistently documented in clinical studies. Indeed, meta-analyses show elevations of CRP, IL-6, IL-12/IL-18, sIL-2R, and TNF-α in MDD. Low-grade inflammation (CRP > 3 mg/L) is present in ~27% of patients, with >1 mg/L in nearly 60%, delineating an “inflamed depression” subgroup [60]. In BD, CRP and TNF-α increase during acute episodes but normalize in euthymia, while IL-6 remains persistently elevated [7]. Transdiagnostically, IL-6, TNF-α, and CRP rise in acute phases of depression, mania, and psychosis, with IL-6 often persisting outside of acute states [61]. Additional hematologic indices (NLR, PLR, and to a lesser extent MLR) also emerge as accessible markers, while immune phenotyping identifies subgroups with neutrophilia, expanded monocytes, CD4+ T cells, and high CRP/IL-6, linked to severity and treatment resistance [62,63]. Key intracellular signaling pathways linking cytokine activity to astrocytic dysfunction are illustrated in Figure 3.

#### 3.2.2. NOD Receptors, NLRP3 Inflammasome, and Pyroptosis

In parallel to surface receptors, additional PRRs are located within the cytosol, where they sense intracellular danger signals. NOD1/2 receptors detect bacterial peptidoglycans that can translocate into systemic blood under conditions of gut barrier disruption [64]. Their engagement provides a priming signal for transcriptional upregulation of inflammasome components through NF-κB activation [19]. A second level of intracellular sensing involves the NLRP3 inflammasome, a multiprotein complex that responds to cellular stress signals such as mitochondrial dysfunction, ROS accumulation, or extracellular ATP. Full activation of NLRP3 requires this dual input: PRR-mediated priming plus an intracellular danger signal. Once assembled, the inflammasome promotes cleavage of pro–IL-1β and pro–IL-18 into their active forms, driving pyroptotic cell death and amplifying inflammatory cascades [19]. Preclinical studies show that chronic stress enhances NLRP3 activation in the hippocampus and prefrontal cortex, leading to increased IL-1β release, inhibition of neurogenesis, disruption of synaptic plasticity, and the emergence of depressive-like behavior [65]. Conversely, both genetic deletion of the NLRP3 gene and pharmacological inhibition of the NLRP3 inflammasome with MCC950 have been shown to reverse these effects, restoring neuroplasticity and behavioral resilience in animal models [66]. Furthermore, conventional antidepressants reduce NLRP3 activation and IL-1β release in experimental models, suggesting that part of their therapeutic effects may be mediated through modulation of innate immune–inflammatory pathways [67]. Conventional monoaminergic antidepressants (SSRIs/SNRIs/TCAs/MAOIs) enhance synaptic serotonin and norepinephrine and, with chronic administration, promote downstream plasticity adaptations; however, clinical improvement typically unfolds over weeks rather than days, reflecting an indirect mechanism of action [68]. Despite proven efficacy versus placebo and broadly comparable effectiveness across agents, outcome heterogeneity is high [69]. In routine care, only about one-third of patients remit at the first adequate trial, with diminishing returns across subsequent steps [70]. Importantly, baseline inflammatory burden is associated with poorer antidepressant outcomes, underscoring a biology not fully addressed by monoaminergic modulation [71]. Emerging stratification data indicate that CRP can inform drug choice: CRP < 1 mg/L predicts better response to the SSRI escitalopram, whereas higher CRP favors noradrenergic/dopaminergic options (e.g., nortriptyline or bupropion-containing regimens) [72]. Collectively, these findings frame conventional agents as necessary but insufficient for biomarker-enriched subgroups, supporting precision approaches that integrate immune–metabolic targets alongside monoamines. Thus, intracellular sensing through NOD receptors and the NLRP3 inflammasome represents a complementary route to TLR4, linking microbial translocation and cellular stress to neuroinflammatory processes in mood disorders [19,20].

### 3.3. Final Common Pathways: Mitochondria, Glia, Plasticity, and Kynurenine Metabolism

The downstream consequences of peripheral triggers (Section 3.1) and intracellular signaling pathways (Section 3.2) converge on a set of final common biological hubs that define the so-called “inflammatory depression” phenotype [25]. These hubs include mitochondrial dysfunction and oxidative stress, glial activation and impaired neuroimmune homeostasis, neurotrophic and synaptic alterations, and dysregulation of the kynurenine pathway. Collectively, they translate molecular immune activation into functional and structural brain deficits observed in MDD and BD [24,73]. Their interplay explains clinical dimensions such as fatigue, cognitive impairment, anhedonia, and reduced responsiveness to conventional treatments.

#### 3.3.1. Mitochondrial Dysfunction and Oxidative Stress

Mitochondria are critical integrators of immune and metabolic stress. Pro-inflammatory cytokines such as TNF-α and IL-1β impair the electron transport chain, reducing ATP synthesis and enhancing ROS production, which further activate the NLRP3 inflammasome and amplify IL-1β/IL-18 release [21,43]. This creates a feed-forward loop between oxidative stress and immune activation. Beyond energy production, mitochondrial dysfunction alters calcium buffering and apoptotic signaling, thereby increasing neuronal vulnerability.

Clinical studies consistently show elevated peripheral markers of oxidative stress in mood disorders, including malondialdehyde (MDA), 8-hydroxy-2′-deoxyguanosine (8-OHdG), and isoprostanes, alongside reduced antioxidant defenses such as total antioxidant capacity, uric acid, and zinc [24,25]. A meta-analysis by Liu et al. (2015) demonstrated increased oxidative damage markers and decreased antioxidants across 115 studies, with partial normalization after antidepressant treatment [24]. Translational interventions support the therapeutic relevance of targeting redox balance [24]. Berk et al. (2008) showed that NAC supplementation replenishes glutathione and improves symptoms, findings subsequently extended to mood disorders in smaller trials [17,74]. Complementary evidence supports mitochondrial-targeted compounds such as omega-3 fatty acids, which exert both antioxidant and anti-inflammatory effects, and creatine, which enhances bioenergetics. Altogether, mitochondrial dysfunction links systemic inflammation to fatigue, psychomotor slowing, and poor treatment response, highlighting a tractable therapeutic axis.

#### 3.3.2. Glial Activation and Neuroinflammation

Microglia and astrocytes form the primary immune regulators of the CNS22,23. Under chronic inflammatory stimulation, microglia polarize toward an M1-like pro-inflammatory phenotype, releasing IL-1β, TNF-α, glutamate, and ROS, thereby contributing to excitotoxicity and synaptic pruning [75,76,77]. At the same time, the reparative M2-like phenotype, normally associated with neurogenesis and tissue repair, is blunted in mood disorders. Yirmiya et al. (2015) reviewed how such dysregulation contributes to depressive phenotypes [23]. Astrocytic dysfunction is equally important: reduced expression of glutamate transporters (EAAT1/2) impairs clearance of excitatory neurotransmitters, while postmortem studies show reduced GFAP+ astrocytic density in corticolimbic regions of depressed patients [22]. These changes reduce trophic support, impair synaptic homeostasis, and amplify excitotoxic cascades. In vivo evidence from TSPO-PET imaging corroborates these findings. Setiawan et al. (2015) showed elevated TSPO binding in the anterior cingulate and other limbic regions [78]. A meta-analysis by Eggerstorfer et al. (2022) confirmed increased TSPO binding in ACC, hippocampus, and insula [36]. However, not all interventions targeting glial activation have proven effective. For example, Attwells et al. (2021) found no reduction in TSPO binding after minocycline, highlighting the complexity of central immune modulation [79]. By contrast, Raison et al. (2013) demonstrated antidepressant effects in patients with baseline CRP > 5 mg/L [80]. Together, these data indicate that glial alterations are central to inflammatory depression but require biomarker-driven therapeutic strategies.

#### 3.3.3. Neurotrophic and Synaptic Alterations

Inflammatory cytokines such as IL-1β, TNF-α, and IL-6 impair neuroplasticity by reducing BDNF transcription and TrkB activation [27]. This leads to dendritic spine loss, reduced synaptic density, and impaired long-term potentiation. Postmortem studies confirm downregulation of BDNF and TrkB expression in corticolimbic areas of depressed and suicidal patients [26]. Peripheral BDNF levels are likewise decreased in mood disorders. A meta-analysis by Fernandes et al. (2015) found reduced serum BDNF across illness phases of BD, with stronger reductions during acute episodes [27]. From a therapeutic standpoint, the restoration of BDNF signaling is essential. Duman et al. (2016) highlighted how ketamine enhances BDNF release and TrkB signaling, underlying its rapid antidepressant and pro-cognitive effects [28]. Emerging small-molecule TrkB agonists (e.g., 7,8-dihydroxyflavone) have shown antidepressant-like activity in preclinical models [81]. Beyond BDNF, other neurotrophins such as vascular endothelial growth factor (VEGF) and nerve growth factor (NGF) may also contribute, although with less consistent clinical data. Overall, impaired neurotrophic signaling represents a convergent hub linking immune stress, cognitive impairment, and treatment resistance.

#### 3.3.4. Kynurenine Pathway Dysregulation

The kynurenine pathway (KP) represents a biochemical bridge between inflammation, neurotransmission, and energy metabolism [29]. Pro-inflammatory cytokines (IFN-γ, TNF-α, and IL-6) induce IDO and TDO, diverting tryptophan from serotonin synthesis toward kynurenine and downstream metabolites [29,31]. This shift increases neurotoxic quinolinic acid (QA), a potent NMDA agonist, while reducing kynurenic acid (KYNA), which normally exerts neuroprotective effects as an NMDA antagonist and α7 nicotinic receptor modulator [32,33].

Meta-analyses consistently support KP dysregulation. Marx et al. (2021) found elevated KYN/TRP ratios, increased QA, and reduced KYNA across mood disorders [30]. Inam et al. (2023) confirmed QA elevations in CSF of MDD patients. Importantly, specific metabolites such as 3-hydroxykynurenine (3-HK) and anthranilic acid contribute to oxidative stress and neuronal toxicity [34].

Clinically, KP alterations are associated with suicidality. Sublette et al. (2011) showed increased plasma kynurenine in suicide attempters versus controls [82]. Additionally, immunometabolic factors exacerbate KP shifts: Agudelo et al. (2014) demonstrated that obesity and insulin resistance enhance IDO activity, linking systemic metabolism to central neurotransmission [83]. Altogether, KP dysregulation provides mechanistic and prognostic insight, suggesting potential for biomarker-guided and precision-targeted interventions.

### 3.4. Region-Specific Neuroinflammatory Changes in Mood Disorders

Neuroinflammation in mood disorders does not occur diffusely but follows regionally selective patterns, affecting circuits that regulate affect, cognition, and motivation. Evidence from postmortem studies, neuroimaging, and recent animal models consistently implicates the prefrontal cortex, hippocampus, amygdala, striatum, and insula. In the prefrontal cortex (PFC), reductions in glial cells—particularly astrocytes and oligodendrocytes—are a robust finding in major depression and bipolar disorder [84]. Recent PET imaging studies show increased TSPO ligand binding in the PFC and anterior cingulate in depressed patients, indicating elevated glial activation in these regions [85].

Chronic stress models also show dendritic spine loss and apical dendrite retraction in medial PFC pyramidal neurons, findings that align with fMRI evidence of impaired prefrontal–amygdala connectivity in inflamed depression [86]. The hippocampus shows strong evidence of neuroinflammatory damage. Microglial activation, astrocyte dysfunction, and downregulation of neurogenesis in the dentate gyrus are reported in both animal models and human studies of major depressive disorder [87,88]. Moreover, recent work shows that hippocampal neuronal survival is decreased, and that treatments targeting microglial polarization (e.g., Urolithin B) can restore neuronal function and reduce depressive behavior [89]. In the amygdala, studies continue to find immune-related modulation. PET and preclinical evidence point to amygdala involvement in mood disorders via increased microglial activation and inflammatory cytokine expression, especially under chronic stress [90]. Reward circuitry, including the nucleus accumbens/ventral striatum, is less well studied in PET recently, but fMRI studies confirm that inflammation correlates with blunted reward response and reduced connectivity [91].

The insula also shows elevated TSPO binding in PET studies of depression [90,92]. Functional imaging suggests altered insular activity during interoceptive and affective tasks in patients with high inflammatory states, consistent with abnormal salience processing.

### 3.5. Individual Differences in Immune Reactivity

Sex, age, and hormonal milieu are key modulators of neuroimmune signaling and contribute to the heterogeneity observed in the inflammation–depression link. Women generally mount stronger innate and adaptive immune responses than men, which has been associated with greater vulnerability to inflammation-related depressive phenotypes [93]. Sex steroids shape these processes in context-dependent ways. Under stable conditions, estrogens and progesterone can reduce microglial activation and dampen inflammatory signaling [94]. However, hormonal fluctuations during reproductive transitions (such as the peripartum and perimenopause) may destabilize neuroimmune balance and increase susceptibility to mood dysregulation. Perimenopause, in particular, is linked to higher rates of depression compared to premenopause, and estradiol withdrawal can trigger symptoms in vulnerable women [95,96,97]. Testosterone is generally associated with anti-inflammatory effects on myeloid cells, including reduced production of pro-inflammatory cytokines, although findings are context-dependent and not fully consistent across immune cell types and experimental settings [98]. Aging represents an additional and robust source of variability. Immunosenescence (decline in adaptive immune competence) and “inflammaging” (persistent, low-grade increases in IL-6, TNF-α, and CRP) create a biological context that enhances vulnerability to late-life depression [99]. Aging-related immune changes have been associated with higher depressive burden and stronger reactivity to inflammatory stressors in older adults [100,101].

Taken together, sex, age, and hormonal status introduce systematic variability in immune reactivity. Considering these biological axes is essential to improve the interpretation of biomarker findings and to refine phenotyping in mood–immune research.

### 3.6. Therapeutic Implications and Stratification Approaches

Translational research strongly suggests that immune–inflammatory mechanisms contribute to mood disorders and may be targeted therapeutically, but effectiveness appears contingent upon biological enrichment [102,103]. Adjunctive celecoxib has demonstrated antidepressant efficacy in MDD and antimanic effects in BD, while infliximab improved symptoms only in patients with elevated baseline inflammation [80]. These findings underscore that anti-inflammatory strategies are not universally effective but may be beneficial in specific subgroups defined by immune activation [60]. Other experimental approaches expand this rationale. Antioxidants and mitochondrial modulators such as N-acetylcysteine aim to counteract the oxidative stress and bioenergetic dysfunction outlined in Section 3.3.1 [17,74].

Alterations in the kynurenine pathway, described in Section 3.3.4, justify growing scientific interest given their link with suicidality and treatment resistance [30,82]. Likewise, the gut microbiome, discussed in Section 3.1.1, has emerged as another axis of translational relevance, with early probiotic and prebiotic interventions showing modest but inconsistent benefits [104].

Together, these strategies highlight that inflammation intersects with metabolic, mitochondrial, and neurotrophic domains, reinforcing the importance of integrated treatment concepts [105]. Clinical stratification efforts mirror this complexity. Biomarkers such as CRP and IL-6, the most robustly replicated findings across studies, have shown prognostic and predictive utility, including differential response to SSRIs versus noradrenergic/dopaminergic antidepressants [72].

Cellular immune phenotyping, neuroimaging evidence of regional neuroinflammation, BBB dysfunction, and multi-omics signatures integrating immune, metabolic, and genetic data further support the operationalization of an “inflammatory biotype” [38,78].

Such stratification is critical to improve reproducibility and translational impact, paving the way for biomarker-enriched and adaptive trial designs. Finally, rapid-acting antidepressants exemplify how mechanistic insights can transform clinical care. Ketamine/esketamine, through NMDA receptor antagonism, glutamate surge, and downstream restoration of BDNF/TrkB signaling, represents the first agent to demonstrate robust, rapid effects beyond monoaminergic modulation [106,107].

**Figure 4:**
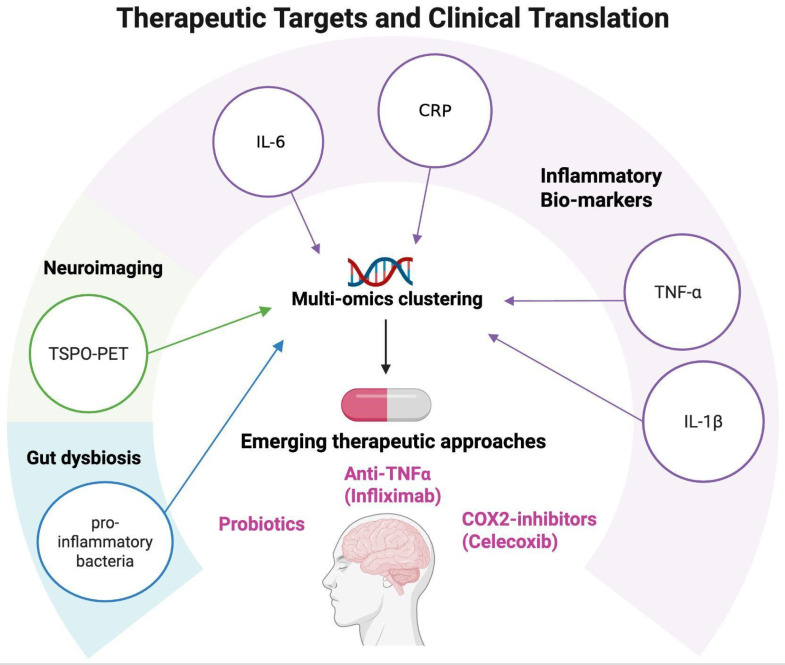
Therapeutic targets and clinical translation. Schematic representation of potential therapeutic targets emerging from neuroinflammation research in mood disorders. Peripheral biomarkers (CRP, IL-6, TNF-α, and IL-1β), neuroimaging markers (TSPO-PET), and multi-omics clustering approaches can help identify patient subgroups with distinct immune–inflammatory profiles. These insights open the way to personalized interventions, including the use of anti-inflammatory agents such as COX-2 inhibitors (e.g., celecoxib), anti-TNF-α treatments (e.g., infliximab), and probiotic interventions, supporting a translational framework for precision psychiatry. Created with BioRender (web-based version). Pinzi, M. (2025) https://BioRender.com/3akca2h (accessed on 14 September 2025).

Collectively, these developments signal a paradigm shift: monoamine dysfunction alone is insufficient to explain treatment response, and future strategies must incorporate immune, metabolic, and neuroplastic dimensions [105]. Potential therapeutic targets emerging from neuroinflammation research are summarized in Figure 4.

### 3.7. Conflicting Clinical Findings and Study Heterogeneity

Despite encouraging signals, clinical translation of immunomodulatory strategies remains inconsistent, and results often diverge across trials [102,108]. For instance, while infliximab produced benefits in patients with CRP > 5 mg/L, it failed to outperform placebo at the group level in treatment-resistant depression [80].

Similarly, meta-analyses of COX-2 inhibitors report beneficial effects in MDD but less consistent findings in BD, with substantial heterogeneity across populations and study designs [109]. These discrepancies indicate that immune-based interventions cannot be considered universally effective, but rather context-dependent [60]. Neuroimaging further illustrates this variability. Some TSPO-PET studies have demonstrated robust increases in microglial activation in corticolimbic regions, whereas others found more modest or null results, likely due to differences in tracers, patient characteristics, and illness stage [36,78].

At the biomarker level, CRP and IL-6 elevations remain the most consistent findings but results for TNF-α and IL-1β are highly heterogeneous, influenced by comorbidities, medication exposure, and methodological variability [105].

Microbiome interventions show inconsistent clinical benefits despite a strong preclinical rationale [104]. These conflicting results highlight two key lessons. First, they underscore that immune dysregulation is not a ubiquitous feature of mood disorders but likely marks a biologically distinct subgroup, consistent with the concept of an “inflammatory biotype” already emphasized in Section 3.6 [38,63]. Second, they emphasize the need for rigorous stratification to separate true therapeutic signals from noise introduced by sample heterogeneity, methodological diversity, and illness-stage effects.

By explicitly recognizing these inconsistencies, the field can avoid premature generalization and instead refine biomarker-guided approaches that integrate immune, metabolic, and neuroplastic domains.

### 3.8. Causality and Directionality of the Inflammation–Mood Link

A central question in immunopsychiatry is whether inflammation causes mood disorders or, conversely, arises as their consequence. Current evidence indicates that the relationship is bidirectional. On the one hand, immune activation can precipitate depressive symptoms. Up to 40–50% of patients treated with interferon-α for hepatitis C or melanoma develop depression, an effect mitigated by prophylactic antidepressants [110,111]. Experimental endotoxin challenge in healthy volunteers similarly induces transient increases in IL-6 and TNF-α, accompanied by anhedonia and dysphoria [112].

Conversely, depression itself promotes inflammatory activity via HPA-axis dysregulation, autonomic imbalance, sleep disturbance, and metabolic changes. Prospective cohorts demonstrate this reverse pathway, with depressive symptoms predicting later increases in CRP and IL-6 [113].

Overall, the most reliable conclusion is that the relationship is cyclical: immune activation can trigger depressive symptoms in experimental and clinical models, while depressive states can amplify inflammatory activity in turn. These reciprocal dynamics, moderated by metabolic and demographic factors, underscore the need for biomarker-guided stratification in future clinical trials. An integrated overview of the evidence discussed in this section is provided in Table 1 (MDD) and Table 2 (bipolar and transdiagnostic studies).

## 4. Conclusions

Mounting evidence supports the existence of an inflammatory biotype in mood disorders, characterized by systemic cytokine elevations, intracellular signaling through NF-κB, JAK/STAT, MAPK, and NLRP3 pathways, and downstream effects on mitochondria, glia, synaptic plasticity, and kynurenine metabolism. Interfaces such as the gut–brain axis and blood–brain barrier provide mechanistic bridges between peripheral immune activation and central neurobiology, delineating a subgroup of patients with greater severity, cognitive impairment, and reduced treatment response. From a translational perspective, these findings highlight both challenges and opportunities. While anti-inflammatory and immunomodulatory interventions have shown promise, their efficacy appears contingent on baseline immune status, reinforcing the need for biomarker-based patient selection. Future research should therefore prioritize the development of standardized biomarker panels that integrate inflammatory, metabolic, and neuroimaging measures, enabling reproducible stratification across cohorts. Large-scale, adaptive clinical trials are needed to test immunomodulatory, metabolic, and microbiome-targeted interventions in biologically defined subgroups. Integration of multi-omics approaches with machine learning and longitudinal designs will be crucial to unravel causal pathways, identify predictive signatures, and refine treatment algorithms. In parallel, novel therapeutic targets—including inflammasome inhibition, mitochondrial restoration, microbiome modulation, and enhancement of BDNF/TrkB signaling—should be systematically evaluated as adjunctive strategies to conventional monoaminergic agents. Collectively, these directions delineate a roadmap toward a precision psychiatry framework, in which mechanistic insights translate into personalized prevention and treatment of mood disorders.

## Figures and Tables

**Table 1:** Evidence on immune–inflammatory dysregulation in major depressive disorder (MDD). This table summarizes 12 studies investigating the role of neuroinflammation in major depressive disorder. The works are grouped by thematic domains (peripheral biomarkers, kynurenine pathway, neuroimaging, oxidative stress, gut–brain axis, and interventional studies). For each study, the experimental design, sample characteristics, key findings, and clinical implications are reported. The overall picture highlights how specific patient subgroups exhibit distinct immune–inflammatory profiles that may guide precision medicine strategies.

Study (Year)	Design	Sample(s)	Key Findings	Clinical Implications
Peripheral Inflammatory Biomarkers and Immune Cell Phenotyping				
Osimo et al., 2020 (Brain Behav Immun) [8]	Meta-analysis	107 studies; 5166 MDD; 5083 controls	CRP, IL-6, IL-12, IL-18, sIL-2R, TNF-α elevated in MDD	Supports inflammatory pathophysiology; biomarkers may aid stratification.
Osimo et al., 2019 (Psychol Med) [60]	Systematic review & meta-analysis	37 studies; 13,541 MDD; 155,728 controls	Low-grade inflammation was prevalent among depressed patients: CRP > 3 mg/L in approximately 27% and CRP > 1 mg/L in about 58% of MDD cases.	CRP screening identifies an “inflamed depression” subgroup.
Lynall et al., 2020 (Biol Psychiatry) [63]	Case–control with clustering	206 MDD; 77 controls	Neutrophils, monocytes, CD4+ T cells, CRP, and IL-6 increased in the inflamed subgroup (~39% of MDD cases).	Biomarker-driven stratification enables targeted immunotherapies.
Kynurenine Pathway & Suicidality				
Sublette et al., 2011 (Brain Behav Immun) [82]	Clinical biomarker study	31 controls; 30 MDD (14 suicide attempters)	KYN elevated in suicide attempters vs. non-attempters/controls;	Elevated KYN linked to suicidal behavior.
Neuroimaging of Neuroinflammation				
Eggerstorfer et al., 2022 (Front Mol Neurosci) [36]	Systematic review & meta-analysis	8 PET studies; 238 MDD; 164 controls	TSPO binding increased in ACC, hippocampus, insula, and PFC.	Direct evidence of neuroinflammation; TSPO as a potential target.
Setiawan et al., 2018 (Lancet Psychiatry) [92]	Cross-sectional PET	MDD patients vs. controls	TSPO-VT shows higher microglial activation in long-untreated MDD cases.	Supports the staging model of progressive neuroinflammation.
Oxidative Stress & Antioxidant Defenses				
Liu et al., 2015 (PLoS ONE) [24]	Meta-analysis (115 studies)	Depression vs. controls; pre/post treatment	MDD is characterized by reduced antioxidant defenses (lower TAC, uric acid, zinc) and increased oxidative damage (elevated MDA, isoprostanes). Antidepressant treatment leads to a partial normalization of these alterations	Confirms oxidative imbalance; supports antioxidant strategies.
Gut–Brain Axis & Microbiota				
Yang et al., 2020 (Sci Adv) [16]	Cross-sectional multi-omics	311 fecal samples (MDD vs. controls)	In MDD gut dysbiosis: increased Bacteroides, decreased Blautia/Eubacterium.	Microbiome–metabolome markers support precision psychiatry.
Wallace & Milev, 2017 (Ann Gen Psychiatry) [104]	Systematic review (10 trials)	Clinical trials	Probiotics improved mood, anxiety, and cognition in most studies; heterogeneity present.	Preliminary evidence for microbiota-targeted interventions.
Skonieczna-Żydecka et al., 2018 (J Clin Med) [15]	Narrative review	Review of studies	Gut dysbiosis linked to depression, anxiety, and FGIDs (functional gastrointestinal disorders).	Supports microbiome-based interventions.
Therapeutic & Interventional Trials				
Raison et al., 2013 (JAMA Psychiatry) [80]	Randomized controlled trial	*n* = 60 TRD outpatients	No overall effect. hs-CRP > 5 mg/L subgroup improved with Infliximab.	Benefit in ‘inflamed’ TRD; utility of CRP cut-offs.
Wang et al., 2022 (World J Clin Cases) [108]	Meta-analysis of RCTs	29 RCTs; 847 depression vs. 810 controls	Celecoxib reduced depression scores; present heterogeneity.	Possible Celecoxib augmentation in selected MDD patients

**Table 2:** Evidence on Immune–inflammatory dysregulation in bipolar disorder and transdiagnostic studies. This table integrates 8 studies, including both disorder-specific evidence in bipolar disorder and transdiagnostic works that jointly investigated MDD, BD, and, in some cases, schizophrenia. The studies cover some of the same thematic areas as in Table 1 (biomarkers, kynurenine pathway, therapeutic interventions, and multi-omics approaches). Together, these findings highlight shared and disorder-specific immune–inflammatory signatures, supporting the hypothesis of common inflammatory pathways and the need for integrative approaches across mood disorders.

Study (Year)	Design	Sample(s)	Key Findings	Clinical Implications
Peripheral Inflammatory Biomarkers				
Solmi et al., 2021 [7] (Brain Behav Immun)	Meta-analysis	49 studies; BD *n* ≈ 1956–2231; controls *n* ≈ 3017–4106	CRP and TNF-α elevated in depressive and manic episodes; IL-6 elevated across states.	IL-6 may be trait-like; CRP/TNF-α more state-dependent
Goldsmith et al., 2016 (Mol Psychiatry) [61]	Meta-analysis	68 studies (acute); 46 studies (chronic)	IL-6, TNF-α, and CRP elevated in acute phases; IL-6 persists in euthymic BD.	Different cytokine patterns across disorders and illness phases; shared biomarkers (e.g., IL-6, CRP) may aid stratification
Kynurenine Pathway				
Marx et al., 2021 (Mol Psychiatry) [30]	Meta-analysis	101 studies; 10,912 participants (MDD, BD, SZ)	TRP and KYN reduced; KA/QA ratio reduced; QA increased (especially in MDD).	KP dysregulation with reduced tryptophan and kynurenine across MDD, BD, and SZ.
Inam et al., 2023 [34] (Braz J Psychiatry)	Systematic review & meta-analysis	23 CSF (Cerebrospinal fluid) studies (MDD, BD, SZ)	In CSF: KA increased in schizophrenia, QA may be increased in MDD, no consistent alterations in BD.	These patterns highlight potential diagnostic and therapeutic implications of kynurenine pathway metabolites, as biomarkers for stratification and possible targets for novel treatments
Therapeutic & Interventional Trials				
Gędek et al., 2023 (J Clin Med) [109]	Systematic review & meta-analysis	44 studies pooled	Celecoxib effective in MDD and mania; no efficacy in bipolar depression.	Antimanic potential; limited effect in bipolar depression.
Multi-Omics & Precision Psychiatry				
Mokhtari et al., 2022 (Prog Neuropsychopharmacol Biol Psychiatry) [38]	Systematic review	—	Potential of integrative omics for biomarker discovery.	Framework for precision psychiatry.
Hagenberg et al., 2025 (Brain Behav Immun) [37]	Clustering (multi-omics)	*n* = 237 individuals	Identified immune-related subtypes with increased CRP, IL-1RA, and CCL2; dendritic-cell dysregulation.	Supports multimodal stratification across MDD/BD.

## Data Availability

No new data were created or analyzed in this study.
